# Gender Harassment Persists in Medical Training

**DOI:** 10.1089/whr.2020.0055

**Published:** 2020-10-08

**Authors:** Carolina Vogel, Theresa Rohr-Kirchgraber

**Affiliations:** ^1^ Marian University College of Osteopathic Medicine, Indianapolis, Indiana, USA.; ^2^ Clinical Medicine and Pediatrics, IU School of Medicine, Indianapolis, Indiana, USA.

**Keywords:** education, gender, harassment, mental health

## Abstract

Medical students start their career with enthusiasm for a profession that emphasizes caring for others so as to promote health, treat injury, and prevent disease. The profession of medicine selects those who have demonstrated compassion, knowledge, and leadership. As students enter the profession, many possess a certain naiveté with the expectation for equity. After a few encounters of her own, one of our authors wondered whether the statistic that female medical students are ∼220% greater to experience harassment in medical education from faculty, staff, and patients was true and sought to determine whether her experience was unique. Unfortunately, with just a quick Instagram post, she received numerous messages from peers indicating that gender harassment is all too common.

## Introduction

In 2018, the National Academies of Science, Engineering, and Medicine released a groundbreaking report on the sexual harassment of women in science, engineering, and medicine. Sexual harassment includes three categories: sexual coercion, unwanted sexual attention, and gender harassment. Gender harassment, the most common form, “refers to a broad range of verbal and nonverbal behaviors not aimed at sexual cooperation but that convey insulting, hostile, and degrading attitudes about ‘members of one gender’.”^1^ Drs Cortina and Jagsi remind us that “gender harassment is hardly benign simply because it is not sexually predatory.”^2^

A lack of understanding that gender harassment has been proven to be harmful may contribute to its continued preponderance in medicine. In a recent study, it was noted that increased experiences of sexual harassment in an academic medical center were associated with lower mental health and job satisfaction, a lack of sense of safety at work, and an increase in turnover intentions.^3^ The consequences of harassment of all forms not only affect the well-being of the individual but also the well-being of the institution.

We start off our medical school experience with some naiveté expecting a level of collegiality and respect. We have chosen this profession presumably for our desire to help others and it is assumed that empathy, compassion, and altruism come with the practice of medicine.

The first time our author experienced gender harassment while a medical student, she wondered, “Did I hear that correctly? Am I oversensitive?” As it happened again and again, she began to identify the experiences as gender harassment. Concerned this pattern was not unique, she reached out to her peers *via* social media.

Utilizing the Instagram story feature on her personal account, our author informed her readers that she was “compiling a list of things she or her peers had experienced in regards to gender bias, racism, xenophobia, etc. in clinical medicine” and posed this question to her followers: “Tell me about an experience you've had with sexism in medicine.” The response from her peers was overwhelming. Within 12 hours >30 responses detailing harassment were sent to the author from her female colleagues who had seen the post.

A common theme among respondents was that they began with a phrase similar to “I'm not sure if this counts but…” Many students were hesitant to label what they had experienced as gender harassment or sexism, though their experiences clearly met the definition of gender or sexual harassment. This phenomenon has been described in previous studies as well.^4^ In all cases where the submitter started with self-doubt, the story that followed most certainly did “count.”

The responses received have been divided into three categories based on content: perceived career advice, female stereotypes, females in allied medical roles, and unwanted sexual attention. Perceived career advice were remarks made by attendings who encouraged female students to pursue primary care because it was assumed that field is more “family friendly.” Some medical specialties remain segregated by gender^5^ and it is important to consider that sexual harassment may play a role in specialty selection of female medical students. In fact, Barbaria documented that “gendered interactions also directly influenced the subspecialty of choice” in their study of Canadian medical students.^4^ In this survey, all responses regarding specialty were instances in which a female student was encouraged to pursue a field that the perpetrator felt was better suited for families. In these instances, it was not only assumed that the female students planned to reproduce but that they had not considered those plans when choosing a subspecialty. These supposedly helpful suggestions undermine both their autonomy and intelligence.

Comments categorized as referring to female stereotypes refer to behaviors or appearances normatively considered female in nature. In one instance, a derogatory “blonde joke” was made, in another a female was told to smile more. These instances of gender harassment are not unique to the medical field and, therefore, draw attention to the pervasiveness of sexism in culture at large. Responses in the allied medical roles category refer to situations in which it was assumed that the female medical student (or in one case, physician) was not acting as a student physician, but as an allied medical professional such as a nurse or patient care technician, presumably because of her female appearance. This assumption is based on an outdated, yet pervasive, stereotype and may be harmful to people of all genders regardless of medical profession. The final category, unwanted sexual attention, was reserved for a singular response to this survey in which a student witnessed her female attending being sexually harassed by a patient. These categories, though not encompassing of all potential forms of sexual harassment, may help readers to identify gender harassment specifically more readily were they to encounter it in the future.

With an interest to explore this experience, an expedited IRB was obtained. All participants were verified as medical students and agreed to have their comments anonymously published.

### Trigger warning: gender harassment

We share these experiences not to shame the perpetrators but to draw attention to the continued pervasiveness of gender harassment in medicine.

## Perceived Career Advice

Male attending: “What specialty are you interested in?”

Female student: “I'm thinking internal med.”

Male attending: “I think you'd be better off with psych, it's much easier to have a family.”

Female student: “I was told I should go into family medicine because it will allow me time off to have a family.”

Female student: “Dr. Smith said I should do surgery as a specialty.”

Male attending: “Really?!” ^*^in disbelief^*^.

Female student: “I want to do family med.”

Male attending: “That's a good idea.”

Female student: “That's a good idea?”

Male attending: “It's better to have a family.”

## Female Stereotypes:

Female student: ^*^answers attending's question correctly^*^.

Male attending: “Wow you're a really good guesser!”

Female student: “I'm not guessing, I just know.”

Male student: “She's a smart cookie doctor.”

Male attending: “It's just remarkable, because you're a blonde.”

Male attending: “You said you're on OB next month?”

Female student: “Yeah, I'm really excited!”

Male attending: “You'll hate it. Every woman I've known that went into OB became a giant bitch.”

Male attending (addressing male student though female student is present): “Be careful about sexual harassment. I've had 3 peers whose lives have been ruined by nasty women saying nasty things about them. And they had wives and children!”

Male attending (briefing female student on the next patient): “This next patient looks great. She's had 8 kids but you'd never even know it!”

Female student: “All of my female standardized patients have given me feedback that I am pretty, but need to smile more. When approaching the instructor, who is a female physician, regarding this, she offered to help coach me when to smile more even after I expressed concern that my clinical skills are not being evaluated.”

## Females in Allied Medical Roles

Male attending to patient: “Let me introduce you to my medical student for the month, Vanessa^*^! And yes, haha, they are letting women into medicine now!”

Female attending: “Yeah, they only stock these old scrubs upstairs in the female locker room. It's weird though, because they put the newer ones in the male locker room upstairs. The old ones go in the female… I guess they just assume women aren't doctors!”

Female student: “A patient called out for help from myself and a male student as nurse and doctor (respectfully… he just needed help walking into clinic). Of course, we went to help and while I was trying to come up with something polite to say once we reached the door, my male colleague said “She's going to be a doctor too and she's smarter than me.” So thankful for that student for having my back.”

Female student: “I'm in medical school!”

Patient: “Oh, are you studying to be a nurse?”

Female student: “No, I'm in medical school. I'm going to be a doctor.”^†^

Many of the experiences included previously were situations in which the perpetrator was an established physician tasked with teaching the female student how to practice medicine. Inarguably there is a power imbalance between medical students and attending physicians as both their grade and future career lie in the evaluation of their performance by the attending. This imbalance places medical students in a particularly vulnerable position when it comes to gender harassment and likely contributes to the lack of formal reporting.^6^ In addition, it is speculated that lack of institutional response to formal complaints contributes to future and continued lack of reporting.^4^ Medicine is a historically hierarchical field and students may fear retaliation. This leads many to believe that reported rates of gender harassment are lower than the actual frequency, and a recent University of Michigan Medical School study's findings suggest that the actual prevalence of harassment is higher than estimated in earlier studies.^3^

Unfortunately, our female medical students are facing gender harassment not just from their educators but also from their patients. However, if physicians are perpetuating gender stereotypes and sexism even in their teaching practices, how could we expect anything less from the patients they are treating?

Trigger warning: gender harassment and sexual harassment: unwanted sexual attention.

## Unwanted Sexual Attention

Female attending: ^*^Performing rectal exam on male patient while female student observes^*^.

Male patient: ^*^Makes comment about how attractive female physician is as her finger is in his rectum^*^.

A lack of understanding of the definition of gender harassment may contribute to the continued pervasiveness of sexism. In fact, the second recommendation of the National Academy of Sciences, Engineering, and Medicine report was to specifically address gender harassment since it is by far the most common form of sexual harassment.^1^ Our author found this to be true of the experiences submitted by her peers as well. The harassment they faced was overwhelmingly gender harassment.

When the NASEM report first came out, many expressed shock that such a high percentage of women, 48%, had experienced sexual harassment. The NASEM report found that female medical students were 220% more likely than students from non-STEM disciplines to have faced sexual harassment from faculty or staff.^1^ This landmark report coinciding with the #MeToo movement and the continued public discussion of women's equality offered hope to many that the culture of sexual harassment in medicine would begin to change. But, our authors question, has it?

Drawing attention to continued sexual and gender harassment in medical education allows perpetrators to recognize their biases and work to change their behavior. Increased self-awareness is an important first step to changing the culture of medicine, although it may not be enough.^7^

We need a robust complaint process with graduated serious consequences. Just as in testing and tracing for a public health crisis, we need to determine the source of the problem to be able to eradicate it. If students are perpetuating gender harassment, they need to be counseled and subsequently released if they cannot reform. Faculty who have had complaints submitted against them must be counseled regarding each complaint, continue to receive graduated consequences when sexual or gender harassment is identified, and be fired if the problem continues. Medical education institutions must support more serious consequences for harassment complaints and more robust reporting protocols.

The NASEM report called academic institutions to action. It is time. We add our voices to the suggestions laid forth in the NASEM report and urge medical training institutions to take tangible steps addressing the culture of harassment in science and medicine. Every medical student should have antidiscrimination training that includes implicit bias training on gender-based discrimination of which harassment is a subset. Medical schools, and their associated institutions, need to educate faculty as well and not just on-line training. With increased diversity training, the establishment of diversity offices, and an anonymous reporting system that allows learners and colleagues to safely report harassment, we begin to address the gender harassment and work to eliminate it.

## Notes

^*^ Psuedonym used for confidentiality.

^†^ The final experience mentioned, where a patient or even a stranger sees a young woman in medical attire and assumes she is going to be a nurse, was by far the most prevalent experience shared by submitters.
